# Evaluation of head orientation and neck muscle EMG signals as three-dimensional command sources

**DOI:** 10.1186/s12984-015-0016-6

**Published:** 2015-03-05

**Authors:** Matthew R Williams, Robert F Kirsch

**Affiliations:** Department of Biomedical Engineering, Case Western Reserve University, Cleveland, Ohio; Cleveland FES Center, Cleveland, Ohio; Louis Stokes Cleveland VA Medical Center, Cleveland, Ohio; MetroHealth Medical Center, Cleveland, Ohio

**Keywords:** User interfaces, Spinal cord injury, Electromyography, Interface evaluation, Command sources, 3D

## Abstract

**Background:**

High cervical spinal cord injuries result in significant functional impairments and affect both the injured individual as well as their family and care givers. To help restore function to these individuals, multiple user interfaces are available to enable command and control of external devices. However, little work has been performed to assess the 3D performance of these interfaces.

**Methods:**

We investigated the performance of eight human subjects in using three user interfaces (head orientation, EMG from muscles of the head and neck, and a three-axis joystick) to command the endpoint position of a multi-axis robotic arm within a 3D workspace to perform a novel out-to-center 3D Fitts’ Law style task. Two of these interfaces (head orientation, EMG from muscles of the head and neck) could realistically be used by individuals with high tetraplegia, while the joystick was evaluated as a standard of high performance. Performance metrics were developed to assess the aspects of command source performance. Data were analyzed using a mixed model design ANOVA. Fixed effects were investigated between sources as well as for interactions between index of difficulty, command source, and the five performance measures used. A 5% threshold for statistical significance was used in the analysis.

**Results:**

The performances of the three command interfaces were rather similar, though significant differences between command sources were observed. The apparent similarity is due in large part to the sequential command strategy (i.e., one dimension of movement at a time) typically adopted by the subjects. EMG-based commands were particularly pulsatile in nature. The use of sequential commands had a significant impact on each command source’s performance for movements in two or three dimensions.

**Conclusions:**

While the sequential nature of the commands produced by the user did not fit with Fitts’ Law, the other performance measures used were able to illustrate the properties of each command source. Though pulsatile, given the overall similarity between head orientation and the EMG interface, (which also could be readily included in a future implanted neuroprosthesis) the use of EMG as a command source for controlling an arm in 3D space is an attractive choice.

## Background

High cervical spinal cord injuries result in significant functional impairments and affect both the injured individual as well as their family and care givers. There are an estimated 250,000 individuals with spinal cord injury (SCI) in the U.S., with approximately 11,000 new occurrences each year [1]. Of this population, roughly 18% are classified as having high-tetraplegia (spinal cord injury at cervical levels 1 to 4) with significant impairment from the shoulders and below [1]. To help restore function to these individuals, multiple user interfaces are available to enable command and control of external devices. The aim of this paper is to investigate the relative performances of several voluntary actions available to an individual with a high cervical SCI that could be used to command the position of a point in three-dimensional space.

Currently, object manipulation in this population can be restored in two ways – service robots or through Functional Electrical Stimulation (FES). Service robots range from workstation based devices (Such as the Handy I [2]), to wheelchair mounted arms like the MANUS [3], to semi- or fully autonomous mobile robots [4,5], acting as “mechanical care givers” reaching, grasping, and moving objects as commanded by their user [3,6]. These robots replace the function of the user’s paralyzed arm and can assist in performing a variety of activities of daily living [3]. It has been found that the use of a service robot can save approximately 2 hours of care-giver time per day in individuals with upper extremity impairment as the user can more readily perform simple tasks independently [7].

Functional Electrical Stimulation is another approach for restoring upper limb function in individuals with a SCI [8] that uses stimulation of paralyzed muscles below the level of injury in a coordinated manner to produce functional movements such as grasping objects [9] and reaching [8]. The use of FES for restoration of hand grasp in individuals with C5-C6 tetraplegia (i.e. individuals that can move their arms, but have no voluntary hand function) has been shown to provide significant improvements in functional ability, independence, and overall quality of life [9,10]. Recent work by Kirsch [11] is developing a FES system to restore full arm function in addition to hand grasp to allow individuals with high tetraplegia to regain some degree of arm movements.

One challenge that both of these solutions share is the need to specify (or command) a point in the workspace where the user desires the end-effector (whether it be a robot gripper or their hand) to be appropriately placed to perform a desired task. Service robots use a variety of means to control the end-point from conventional user interfaces (joystick, keyboard, or 3D mouse) [6,7,12], voice commands [6], graphical user interfaces (either as a keyboard as in [13] or along with object recognition [12]), touch-screens [5], or laser pointers [5]. Many of these interfaces are either not applicable to individuals with tetraplegia because they cannot be operated due to the paralysis or require a complex stereo-camera system attached to the wheelchair, looking over the user’s shoulder and an onboard computer for image processing [5,12]. Other means to command a point in space come from the area of virtual reality and other 3D computer interfaces, such as the Space Mouse or optical-based gesture recognition, but these approaches require the use of the hands and are thus not applicable to individuals with high tetraplegia.

Based on the requirements of the SCI population, the user interface for controlling a portable 3D device (rehab robot or FES system) must 1) remain under voluntary control following high cervical spinal cord injury (C3) or lower, 2) not interfere with common activities of daily living such as eating, grooming, or conversing, 3) not encumber the user with an over abundance of worn equipment, 4) be portable on a conventional electric wheel-chair in terms of size and weight, and not require sensor arms extending from the chair, rear-facing cameras or other apparatuses that would interfere with common activities such as sitting at a restaurant table or using public transportation, 5) use small, discrete, or preferably unseen sensors, and 6) be easy to activate and operate by a lay-person (non-clinician or technician) and their care-givers.

One emerging field that does meet many of these criteria is that of brain-computer interfaces (BCIs). While BCIs do represent an intriguing potential new user interface for individuals with severe neurological deficit, they are not yet proven as a clinically viable alternative given the amount of equipment, technical support, and system training involved [14]. However, BCI approaches are often focused on the same user interface need, and their performance in this role should be evaluated using the same types of metrics described here.

The first quantification of human motor performance was in 1954 by the work of Fitts [15]. Based on Shannon’s original work in communication theory [16], the Fitts study showed that any motor task conveys a finite amount of information that is limited by the capability of the system to perform a task both rapidly and accurately. The tradeoff between these two aspects of motion is a relationship that became known as Fitts’ Law. The same relationship has been found to hold for human commanded cursor motion whether using computer mice, joysticks, cursor keys [17,18], or trackballs [18]. While a number of conventional 2D computer interfaces have been critically and quantitatively evaluated, little rigorous assessment has been performed on 3D user interfaces. The robotic control studies described above typically used a timed functional task (e.g., the time required to pick up an object) as the performance metric. In the case of VR interfaces, the task is often navigating a maze [19,20]. These evaluations are often focused on the robot or VR system *per se* rather than on the performance of the command interface. Two studies that have critically analyzed 3D input devices primarily measured the tracking performance of a moving virtual object. The work of Klocheck [21] compared the performance of a standard mouse to a game-pad in tasks related to “first person shooter” video games, with a number of time-based performance measures related to target acquisition, tracking, and leading. A study by Zhai [22] investigated the effects of coordination of control of multiple degrees of freedom on the performance of a 6 DOF input device in a docking task using both time and movement efficiency as performance measures. Other than these, little other quantitative analysis has been performed on 3D user interfaces to command motion through a workspace. In particular, no interfaces that would be usable by individuals with a high cervical spinal cord injury have been evaluated.

This study developed a quantitative 3D command source analysis tool and used it to compare the performance of two command interfaces (head orientation and the EMG of the muscles of the neck and face). Both of these interfaces are somewhat artificial for controlling arm-like movements, as neither has a physical relationship to arm movements. Although neither of these user interfaces are optimal for controlling arm movements, they are one of the very few interfaces currently available to individuals with high tetraplegia for controlling an object in 3D space (i.e., the end-point of a robot arm). Specifically, both of these actions remain under voluntary control following a high cervical spinal cord injury (C2 and below), and execution of these actions do not significantly interfere with activities of daily living such as eating, grooming and communication [23]. As a standard of comparison, we also evaluated the performance of a three-axis joystick. This is an appropriate standard because joystick control is also somewhat physically unrelated to the whole arm movements we intend to control, but it does engage some upper extremity motions and is likely to be slightly more intuitive to the users. The study explored the performance of these three 3D interfaces across 1, 2 and 3 dimensions, and expanded upon previous work by the authors [24] which investigated similar command sources as 2D user interfaces.

## Methods

**Figure 1:**
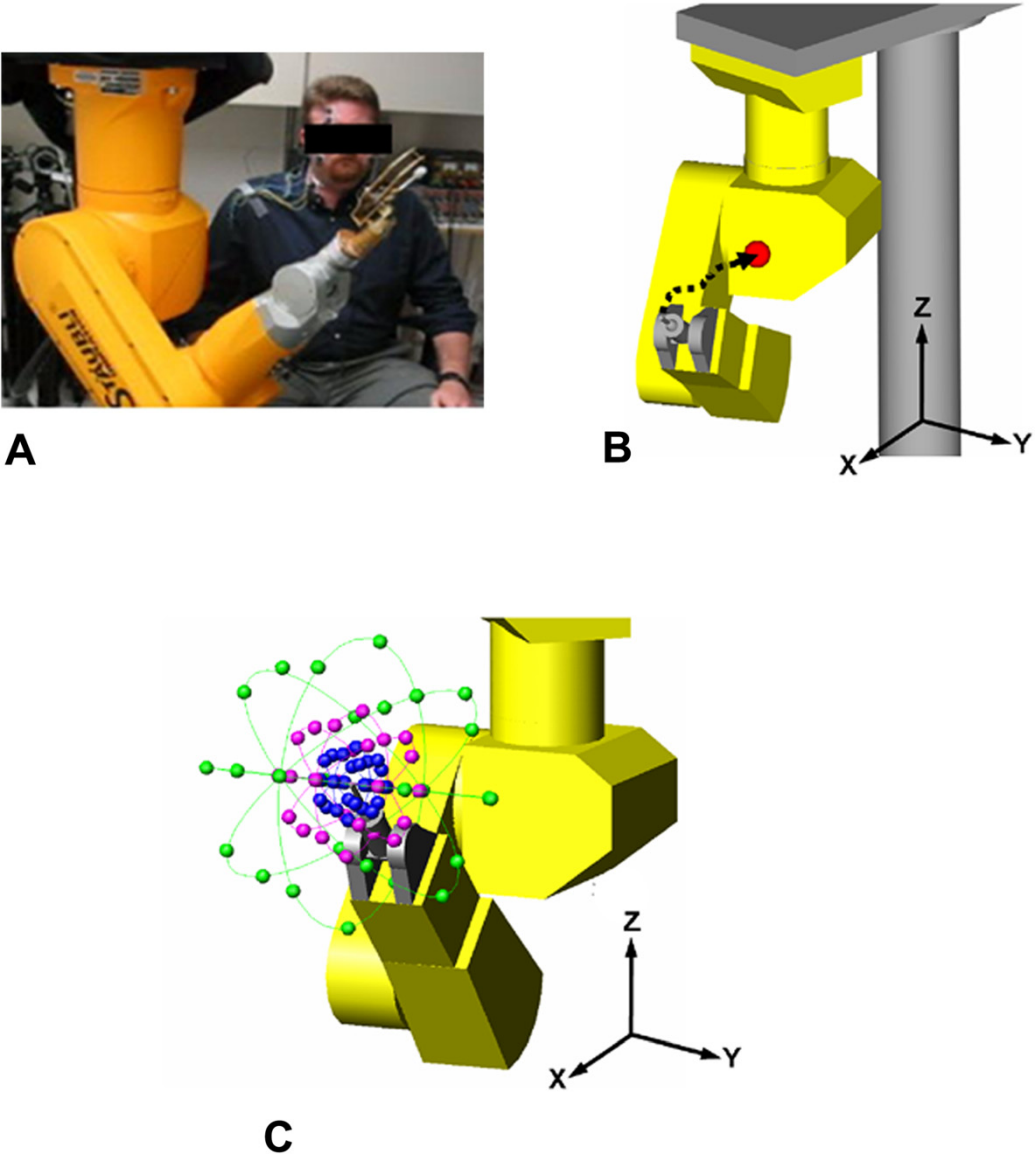
**Robot used in experiments. (A)** Photograph of subject seated next to robot arm. **(B)** Example of typical out-center task. **(C)** Rendering of all starting locations with end-point in the center location.

Subjects performed an inverted Fitts’ Law style “out-to-center” task, commanding the robot to move to a common center point from a number of randomly presented starting locations located in various directions within a 400 mm diameter sphere centered in front of the subject (Figure 1A). Movements were commanded using one of three command inputs (head orientation, EMG, or three-axis joystick) via custom control software implemented under Matlab (The MathWorks, Inc.). Performance was assessed using seven metrics related to movement accuracy, stability, and speed. These techniques are detailed in the following sections.

### Task

A novel out-to-center task was employed (Figure 1B), with a single final target location and starting locations radially distributed in 26 directions and at three distances (50, 100, and 200 mm) from the center (target) location. In addition, three different target widths (25, 50, and 100 mm) were used, for a total of 156 out-to-center trials. As target width could not be greater than target distance, the shortest distance (50 mm) used only the smallest targets (25 mm), the middle distance (100 mm) used the two smaller target sizes (50 and 100 mm), while the longest distance (200 mm) used all three target widths. The out-to-center task was used because it simplifies target presentation in that only a single central target needs to be presented, while the effect of various directions of movement can be evaluated by varying the starting location. Starting locations were presented in random order within the 400 mm sphere centered approximately 500 mm in front of the subject. This volume represents the expected reaching space of an individual with a high cervical SCI using an assistive device. Locating the center point at 500 mm in front of the participant kept the endpoint of the robot a comfortable distance from the face and torso of the participant while still keeping the farther starting location within reach of the robot. Starting locations were located on 26 different 45° radial lines, as illustrated in Figure 1C. A Cartesian coordinate system was used as it was found to yield the best performance (compared to cylindrical or spherical coordinate systems) in initial pilot experiments as it was the most intuitive for users to control.

A two second target dwell time within the target volume was used to indicate successful target acquisition. Upon target acquisition the subject was alerted by a buzzer and the robot arm was automatically moved to the next starting location after a 1 second pause. Upon reaching the new starting location, the trial was initiated and the user alerted with a buzzer to commence a new target acquisition. This task included a wide range of movement directions and, depending on the spatial relationship between the starting position and the center point, required the users to give commands for 1D, 2D, and 3D motions of the robot endpoint. This experimental structure was well tolerated by our subjects in terms of attention requirements and experiment duration. Each command source was tested over three blocks of 156 starting locations and lasting approximately 45 minutes each, with a five minute break between blocks.

The target was indicated by a physical, open-framed sphere suspended from the ceiling and subjects commanded the robot end-point into the space defined by the target sphere. Spherical targets were used to eliminate the effects of approach angle on target width, similar to the use of use of circular targets in 2D human-computer interface studies [25]. The Shannon form of the Index of Difficulty (Equation 1) was used to compare different combinations of target distance and width, consistent with current literature and human-computer interface evaluation standards [25].

1 $$ \mathrm{ID}=lo{g}_2\left(\frac{\mathrm{D}}{\mathrm{W}}+1\right) $$

Table 1 details the distances, widths, and corresponding Indices of Difficulty (ID) used in the experiment. Three different distances (D) and target sizes (W) were used for a total of 9 distance-target size combinations that produced Indices of Difficulty ranging from 1.58 to 3.17 bits. The selected distances and target sizes were dictated by the available workspace of the robot arm used in the study.

**Table 1:** **Target widths (W) and distances (D) and the resultant Indices of Difficulty**

	**D (mm)**		
**W (mm)**	**50**	**100**	**200**
**25**	1.58	2.32	3.17
**50**		1.58	2.32
**100**			1.58

Subjects were instructed to move the end-point from the randomly presented starting location to the central target as quickly and accurately as possible. Subjects were not specifically told what speed or path to take, and thus self-selected trajectories and speeds.

### Command sources

**Figure 2:**
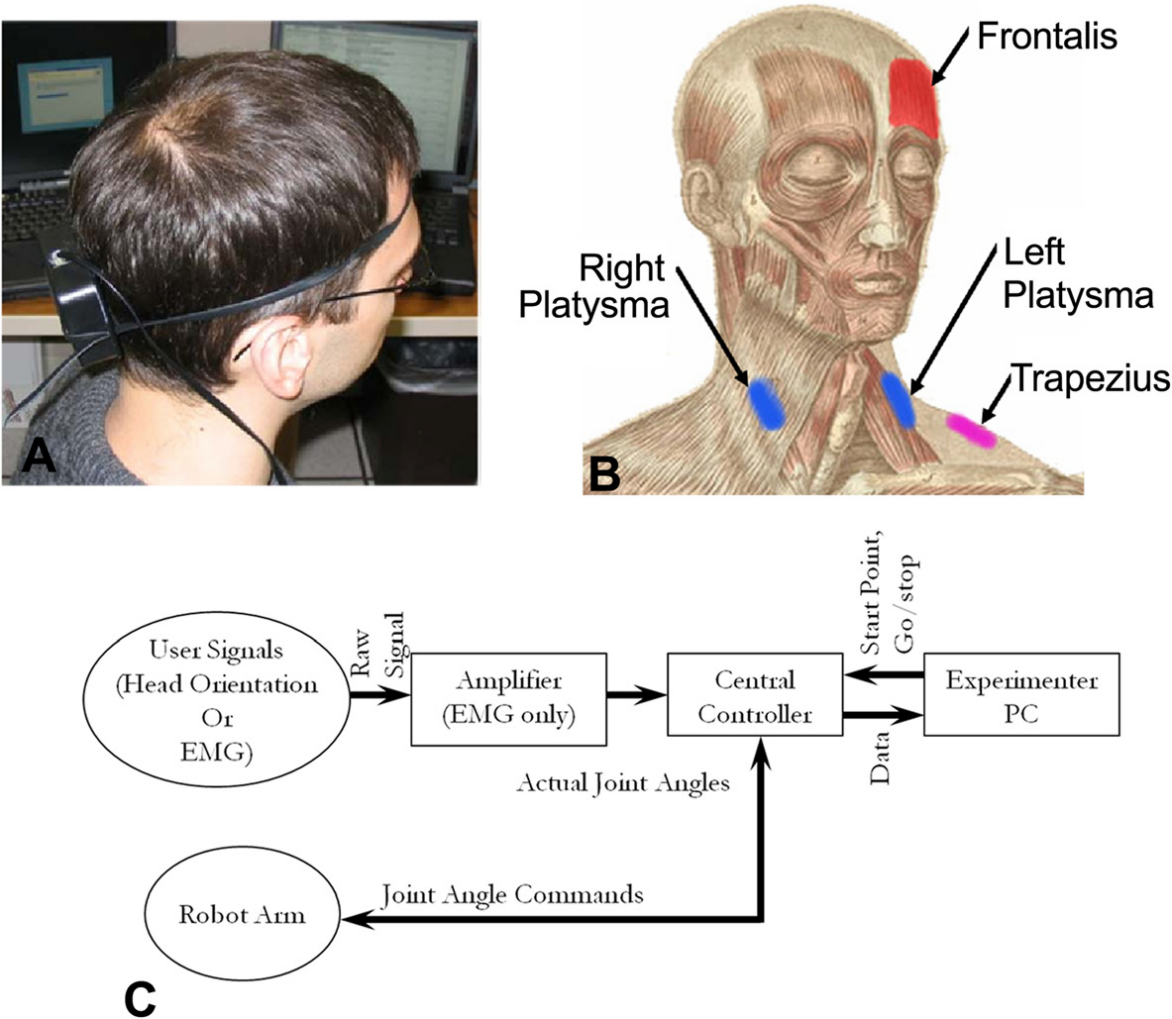
**Command sources and experiment system architecture. (A)** Photograph of subject wearing head orientation sensor. **(B)** Illustration of muscles used for the EMG command source. **(C)** Diagram of the experimental system architecture.

Given the criteria for SCI user interfaces described earlier, three command sources were evaluated: head orientation, EMG of the muscles of the face and neck, and a three-axis joystick (as a control). These command sources (other than the joystick) are useable by individuals with tetraplegia as they are controlled using voluntary actions that are above the level of injury. Head orientation angles were measured using a three degree of freedom orientation sensor (MicroStrain 3DM) attached using an elastic headband to the back of the subject’s head (Figure 2A), similar to other studies [7,12,24]. The sensor did not encumber the subject as its size and weight (89 mm × 64 mm × 25 mm, ~75 g) were small enough to not significantly affect head movements. Head pitch angle was used to command up-down (z-axis) motion of the robot, head yaw angle commanded left-right (y-axis) motion of the robot, and head roll angle was used to command in-out (x-axis) motion of the robot. The sensor communicated with the controller PC hardware via RS-232 serial communication (9600 bps, 8 N1), transmitting a 7 byte packet - 1 status byte and 2 bytes each for roll, pitch, and yaw.

Surface EMG signals were recorded from one facial muscle and three muscles of the neck (Figure 2B) using disposable, self-adhering, pre-moistened silver/silver chloride electrodes. Electrodes were placed on the muscle belly of each muscle used, parallel to the action of the muscle and spaced approximately 40 mm apart (center to center) such that they fit on the muscle but were not touching. Skin surface preparation included a vigorous scrub with an alcohol wipe at each electrode location to remove oil and lightly debride the skin. Once the electrodes were placed, they were further secured to the subject with a strip of surgical tape. Signals were recorded from the left and right platysma (left and right motion commands), the left trapezius (for upwards motion commands), and the frontalis (downwards motion commands). Depth commands (in and out motions relative to the subject) were computed based on co-contraction patterns: simultaneous contractions of the left and right platysma were used to command outward motion and simultaneous contractions of the trapezius and frontalis were used to command inward motion. Subjects were told to contract and maintain the contraction of these muscles and were shown an example of these muscles contracting by the experimenter. Practice to control the contraction of these muscles and their use as command inputs was performed as described later in the Protocol sub-section of the Methods. These particular muscles were selected because they remain under voluntary control following cervical spinal cord injury, generate independent voluntary actions, are in locations that are not subject to significant motion artifact, and are not important contributors to head motions. Four muscles were used as command sources because this is the number of EMG recording channels available in an existing implantable neuroprosthesis for high tetraplegia [11]. We did not investigate alternative muscle sets or include different numbers of EMG signals, because of limitations in experimental session duration, and because preliminary testing indicated that the selected set was more natural and had higher performance than other muscle combinations. Differential, bipolar surface EMG signals were high pass filtered at 0.16 Hz to knock out DC and eliminate most movement artifacts, amplified with a gain of 3300 using CED 1902 amplifiers, anti-alias low pass filtered at 1000 Hz, sampled by an AD converter at 2 kHz, and rectified. These signals were then passed through a 1 Hz digital low pass filter for smoothing and signal amplitudes less than 5% of maximum were set to zero to remove baseline noise. For voluntary contractions, virtually all information is dominated by low frequency components and the 1Hz filter was found to produce a smooth EMG envelope pattern and resulting in a relatively noise free command signal. The 1 Hz low pass filter was used as it was a good compromise between smoothness and responsiveness of the command signal. Higher cutoff frequencies were found in earlier pilot studies to produce unusable velocity command fluctuations that prevented reasonable performance of the task.

The joystick used in this study consisted of a three-axis mounting for the same MicroStrain sensor used to measure head orientation. The same action commands and data transmission described for head orientation above were used with the joystick. The user held the joystick at the top and an elastic mechanism re-centered the joystick when released, providing tactile feedback to the user as to the degree of deviation from center and allowing users to keep their eyes on the workspace. The joystick was used as a control to represent the performance of a 3D command source used by an able-bodied individual by which the command sources intended for use by individuals with SCI can be compared.

### Hardware

The central controller used in the study consisted of a single board computer running a custom control system programmed using xPC Target (The Mathworks, Inc.). This controller (Figure 2C) received and processed signals from the user, converted these into robot end-point velocity commands, and (using an inverse kinematic model), calculated and delivered joint position commands to the robot. The control system operated at 100Hz. The system operated in real-time, with all signal processing and transmission steps occurring during the 10 ms step-time such that no lag in end-point motion was noticeable by the subject. The central controller also received position information back from the robot and transmitted all collected data (user signals, commands, and actual robot position) to a separate PC for later analysis

The Staubli RX-60 robot arm used in this study (Staubli Inc., Pfäffikon, Switzerland) has dimensions similar to those of a human arm. The arm was mounted with the base at the top and the rest of the arm suspended below, with the shoulder located at about shoulder height for a seated individual (Figure 1A). The role of the robot arm was to serve as a proxy for a paralyzed arm actuated by an FES system – similar to a system that could be used by an individual with a spinal cord injury. The robot was programmed to accept joint position commands from the central controller and return actual joint positions. The end-point of the robot was indicated by a pointer attached to the wrist that approximated an extended index finger.

### Velocity algorithm

A proportional velocity-gated ramp algorithm converted user signal levels into end-point velocity commands. Above a command source-specific threshold, user signals were converted into an end-point velocity command that was proportional to the square of the ratio of the amplitude above threshold to the overall range of the user signal, as described in earlier work by the authors The maximum command velocity was set to 300 mm/sa value, (and the range from 0 to maximum speed), that was determined in preliminary testing to be the highest gain that could be commanded in a stable manner to maximize performance while minimizing movement time. For head orientation, the maximum command signal was generated for a neck angle of 30°, such that subjects could still see the entire workspace comfortably. For the EMG command source, the maximum user signal was calculated to be about 70% of the maximum voluntary contraction (MVC) EMG, a level that was repeatable and sustainable for subjects without producing undue fatigue (during calibration, subjects were asked to contract a “moderately strong, but repeatable” amount which generally measured around 70% of their MVC). The joystick used the same 30° limit as head orientation, keeping the required hand motion distances consistent with similar devices.

For head orientation and joystick, the threshold for movement was 10° off-center, which allowed for natural drift within a command “dead zone” [24,26], and accounted for small natural head movements unrelated to controlling the arm and any minor user error in centering the joystick. The threshold for EMG was set at 20% of maximum voluntary contraction (MVC) but also included compensation for changes in electrode impedance due to perspiration over the course of the experiment. A floating threshold algorithm was used to maximize the dynamic range of the EMG signal by adjusting the baseline of the EMG signal every 10 seconds to account for any drift that may occur [24]. These methods and thresholds for movement initiation were based on earlier pilot studies and were found to be “functionally optimal”, reducing effort and *Overshoot* while reducing lag in starting endpoint movement compared to other threshold values. As such, the thresholds selected are a compromise between controllability and swift movement. Lower thresholds often resulted in more unintended movements and increased *Overshoot*, while higher thresholds required greater head movement and a greater amount of muscular effort, reducing overall robot controllability.

### Performance metrics

Several measures of command source performance were used. These include traditional performance metrics used to assess user interfaces, but also include measures that look more closely at the individual performance aspects that affect overall command source performance. This work expands into three dimensions the 2D performance measures previously published by the authors [24].

*Throughput.* The *Throughput* (TP) for each individual target trial was computed by dividing the Index of Difficulty (Equation 1) by the movement time to reach the target for each trial and then averaged across all factors (trials, locations, and indices of difficulty) for each command interface and for each subject [24,25]. This yielded a single value summary of command source performance as it incorporates the individual effects of each aspect of target acquisition (direction, size, and distance) and individual subject differences.

*Path Efficiency* (PE) is a measure of the straightness of the end-point path to the target. It is computed by dividing the straight-line distance by the actual distance traveled.

*Overshoot* is the number of occurrences of the robot end-point being in the target space and then leaving the target before the end of the 2 second dwell time (across all targets), divided by the total number of targets.

*Reaction Time* (RT) is the time between the trial start buzzer and initiation of robot movement commanded by the user. It is a measure of the time the participant takes to recognize the start prompt, plan, and initiate execution of the movement command and is indicative of the “responsiveness” of the user interface.

*Average Speed* is the average non-zero speed of the end-point over the course of the trial.

*Dimension Ratio.* A *Dimension Ratio* (DR) was calculated for each of the three command sources and each of the performance measures described above. This metric is similar to the *Direction Ratio* previously published by the authors [24], but with the expansion of the work to a 3D task. Each DR illustrates the effect of moving in multiple dimensions (which requires the generation of multiple commands simultaneously) versus moving along a single Cartesian axis (which requires only a single command) on the performance measures. It is defined as the average performance when moving in either two or three dimensions divided by the average performance when movement to the target required only a 1D motion (i.e., along the x, y, or z axes). The greater the deviation from unity (identical single dimension and multi-dimension performance) the greater the effect of moving in multiple degrees of freedom has on command source performance. An additional DR is calculated to illustrate how performance is affected when moving in three dimensions compared to two by dividing the average 3D performance by 2D performance.

*X,Y,Z Commands*. We quantified the ability of a command source to provide a smooth, continuous command to the target versus an intermittent, multi-component command by summing the number of times the X, Y, and Z commands transitioned from below to above threshold during the acquisition of a target. The greater the number of commands issued, the more pulsatile the command source, indicating that the command source achieved the target via a sequence of small-amplitude commands, which is presumably less natural and has a higher cognitive burden.

### Protocol

All subjects were recruited from the graduate student population of Case Western Reserve University and were healthy in regards to their ability to voluntarily control facial and neck muscles, head orientation, and operate a joystick. None of the subjects had a spinal cord injury. Given that the command sources to be tested are available and under voluntary control for individuals with an SCI, able-bodied subjects served as an appropriate model for the intended target population. Proper informed consent was obtained and pertinent human subject protections observed, including approval by the Louis Stokes Cleveland VA Medical Center Institutional Review Board. Eight subjects participated in the head orientation and joystick experiments. Of those eight subjects, two were disqualified from the EMG portion of the study because they were unable to independently control the left and right platysma muscles. Thus, data from six subjects were used to summarize EMG-based performance and the performance of all eight subjects was used to assess the head orientation and joystick command sources. Appropriate steps were taken in the statistical analysis to account for the difference in population sizes.

Subject performance using each of the three command sources was evaluated on different days, with each experimental session focusing on a single command source (randomly determined). The experiment consisted of a 5 minute practice with the command source being tested, followed by the three blocks of 156 trials. The first block served as a recorded practice. Blocks 2 and 3 were used to calculate individual subject performance. In preliminary testing, it was shown that while some improvements in performance were seen by some subjects between blocks 1 and 2, no statistical performance difference (p = 0.15, 0.38, and 0.28 for head orientation, EMG, and joystick, respectively) was observed across blocks 2 and 3 indicating a performance plateau. The total length of an experimental session (including set-up, practice, three experimental blocks and rest periods) was approximately three hours.

Overall performance measures (performance across all subjects), across target dimensions (1, 2 or 3 DOF to the target), and across directions (i.e. moving forward vs. backward) for each command source were tested for normality and having been found to fit a normal distribution, were analyzed using a mixed model design ANOVA. Fixed effects were investigated between sources as well as for interactions between index of difficulty, command source, and the five performance measures previously described. A 5% threshold for statistical significance was used in the analysis.

## Results

**Figure 3:**
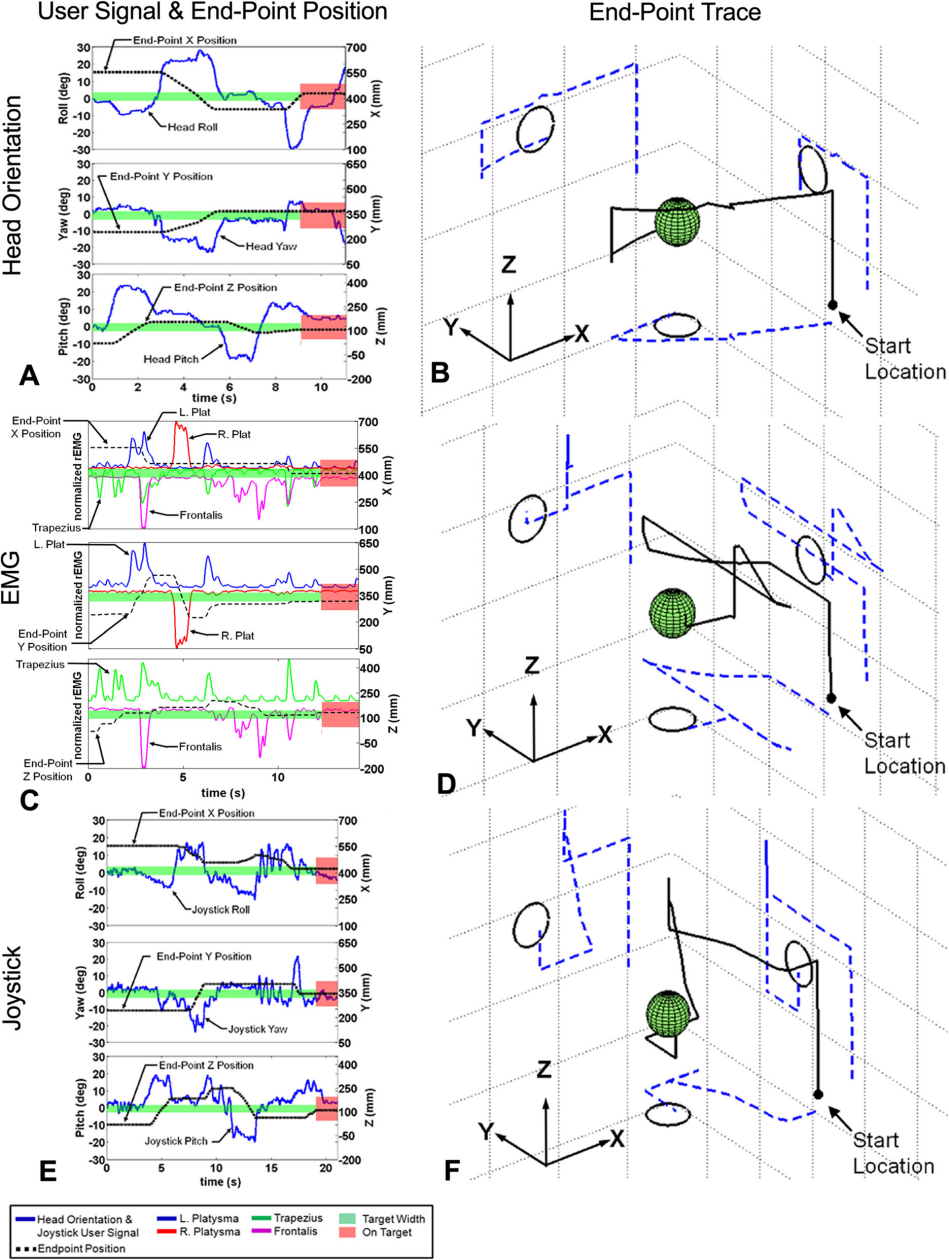
**Example of a single user’s performance from an identical starting location across command sources.** The left column **(A, C, & E)** shows the user signals and resulting end-point position. The highlighted band across the middle of the plots represents the target width (changing color and becoming wider for emphasis when the arm end-point is inside the target space). The right column **(B, D, & F)** illustrates the end-point traces through the 3D workspace. Note that most of the motions are sequential, with few diagonal motions.

Figure 3 illustrates the user commands and resulting 3D end-point trajectories generated by a single subject using different command interfaces to move from the same starting location and ending at the same target. The target width is represented by the shaded bar across the center of each plot (Figure 3A, (C) and (E)). Figure 3(A) and (B) were generated using head orientation command signals, Figure 3C and (D) using EMG command signals, and Figure 3E and F using joystick command signals. In general, commanded motions to 3D targets did not follow straight-line paths (despite being possible with each command source), but rather tended to move along one axis at a time. This can be seen in the lower panels of Figure 3a, (C), and (E) as z-axis (vertical) commands preceding the other two axes, and in Figure 3B, (D), and (F) as initial vertical movement that is then followed by movements long the other two axes. EMG showed the most irregular, intermittent command structure, with more numerous, smaller steps (Figure 3C and (D)) to the target compared to head orientation (Figure 3A and (B)) or joystick (Figure 3D and (E)). The joystick showed some oscillation in the user signal, but because this was primarily below the threshold for activation, it did not significantly affect the number of commands issued (discussed below) or the overall smoothness of the end-point path.

**Figure 4:**
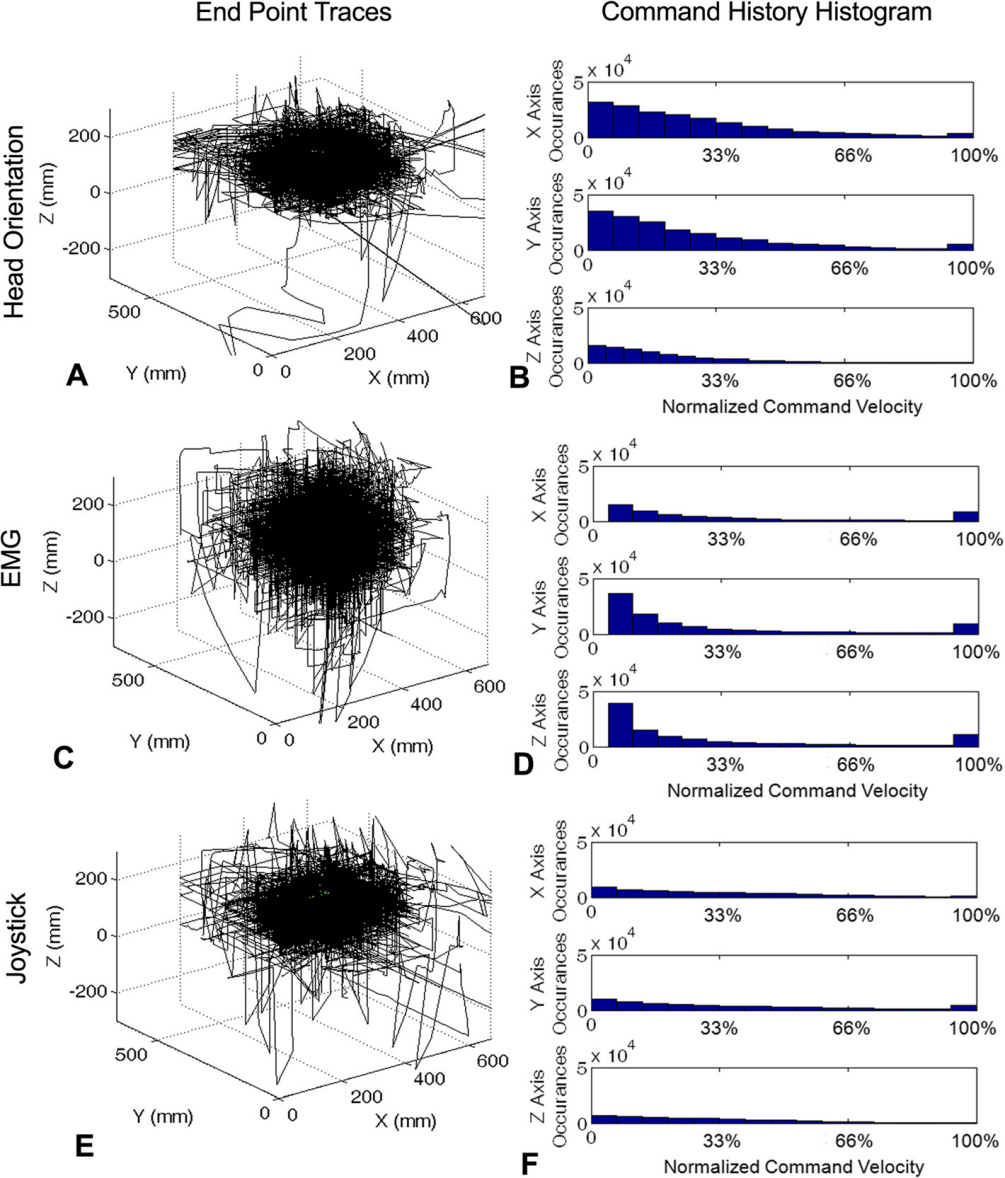
**End-point traces (left column, A C & E) and command velocity histograms (right column, B, D, & F) for all subjects across command sources.**

Figure 4 shows the cursor motions and velocity histograms for all targets and all subjects. Each column indicates a different command source, with the top row showing the end-point movements and the bottom row illustrating the velocity histograms. The head orientation and joystick-commanded end-point movements (Figure 4A and (E)) were more contained within a smaller volume, with a flatter overall profile than that of the EMG end-point traces (Figure 4C). Figure 4B, (D), and (F) show the histograms of the user-commanded velocities for head orientation, EMG, and joystick commands, respectively. These histograms indicate the preferred velocity ranges for each of the command sources. Across command sources, lower velocity commands were more common, with a progressively declining number of occurrences of higher speeds. This trend does not hold for the highest velocity bin, indicating that subjects were somewhat limited (by the maximum robot velocity we set) during certain portions of movement. In Figure 4B, very little speed limiting can be seen in head orientation forward/backward (x-axis) and lateral (y-axis) commands at maximum speed. The histogram of EMG command velocities (Figure 4D) shows some speed limiting across all directions. Figure 4F, the joystick command velocity histogram, shows some speed limiting, in forward/backward (x-axis) and lateral (y-axis) issued commands and none in the vertical (z-axis).

**Figure 5:**
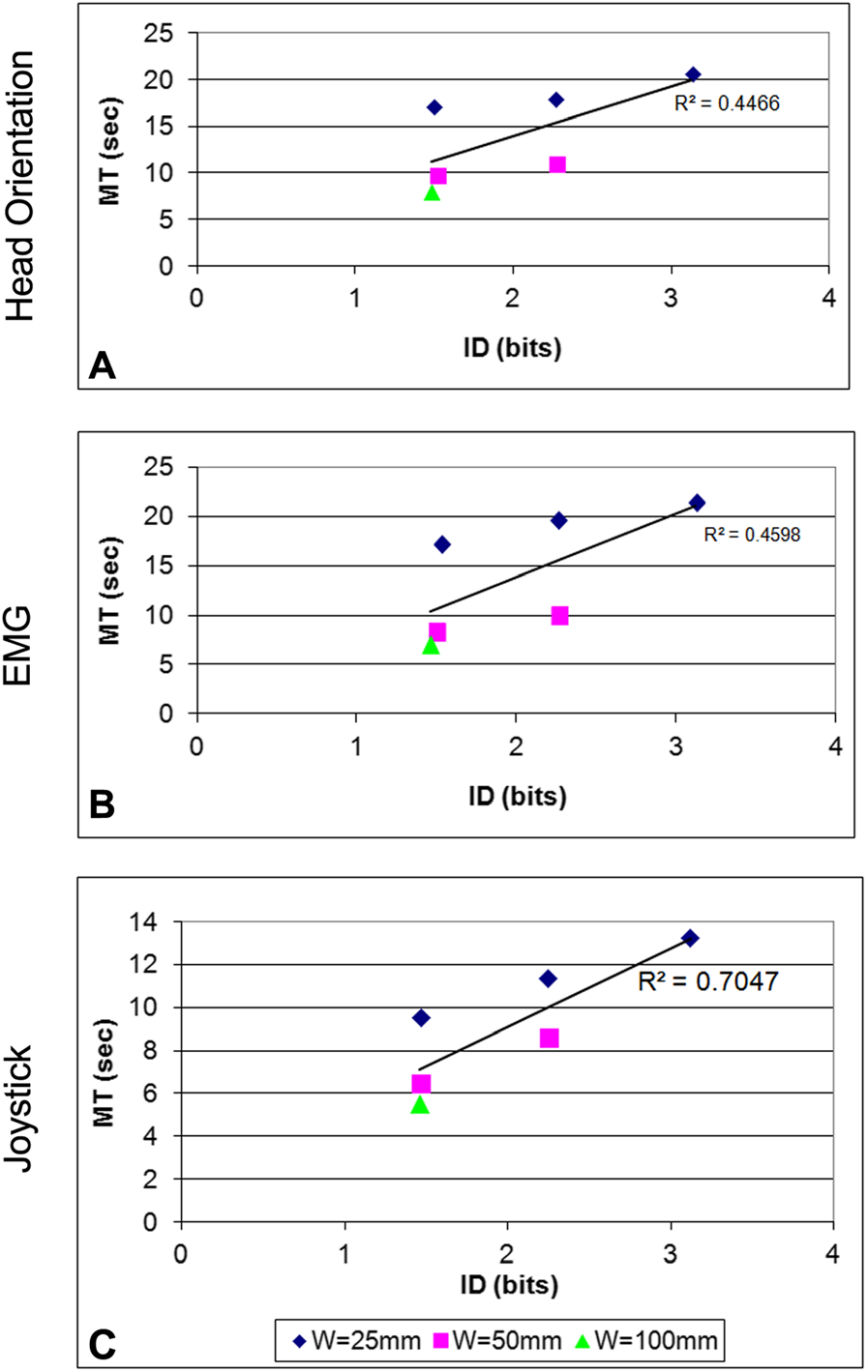
**Regression plots of end-point Movement Time to Index of Difficulty for each command source (A-C).**

Figure 5 illustrates the relationship between movement time (MT) and index of difficulty (ID). For all three command sources, MT increased as the ID increased. Joystick-commanded end-point movements displayed the most linear (i.e., Fitts’ Law-like) relationship (R^2^ = 0.70) between ID and MT (Figure 5C). Head orientation and EMG command sources (Figure 5A and (B)) had lower overall linear correlation between ID and MT (R^2^ = 0.45 and 0.46, respectively), and the relationship depended more strongly on target width (W).

**Figure 6:**
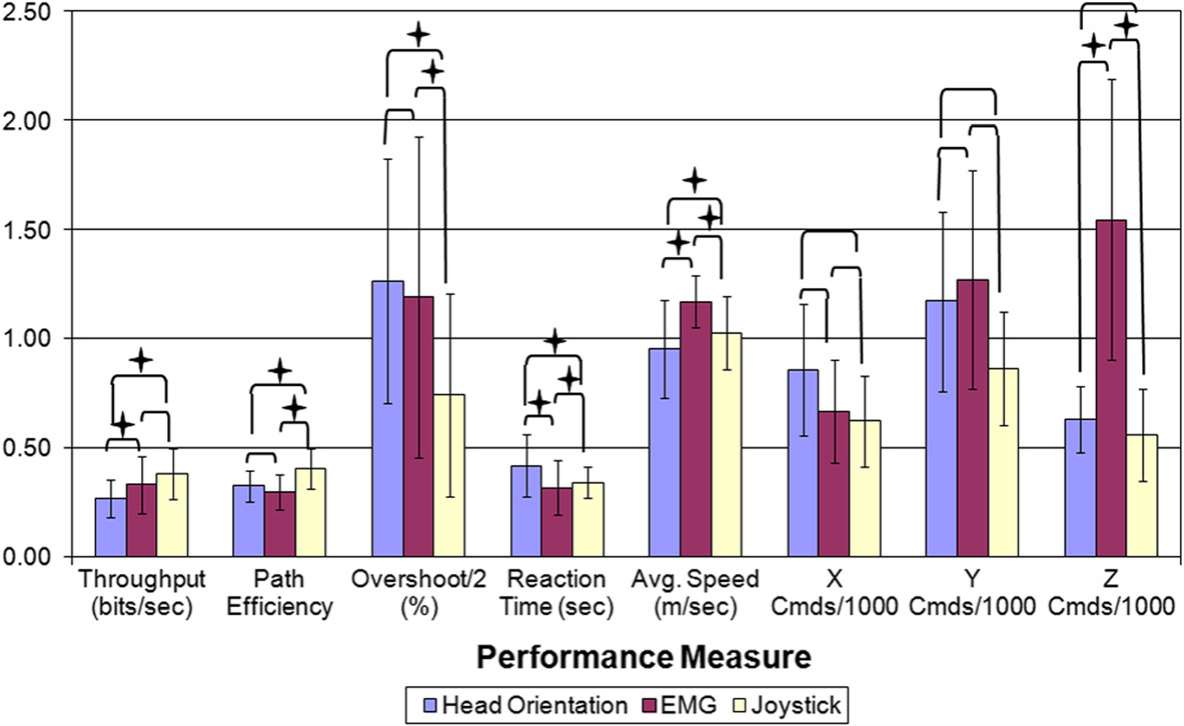
**Comparison of command source performance measures across sources.** Pair-wise comparisons are noted with brackets. Comparisons that are significantly different (p ≤ 0.05) within the marked groups are denoted with a star. Given the overall high level of Overshoot, the Overshoot in this graph is the Overshoot divided by 2 in order to make the magnitude more consistent with the other performance measures presented in the figure.

Figure 6 and Table 2 summarize the performance of each command source across the various performance metrics. For all comparisons, statistically significant differences were seen across all performance measures (p ≤ 0.0048 or less). Head orientation had a significantly lower *Throughput* than EMG (p = 0.004) while no difference was observed between EMG and the joystick (p = 0.122). No significant difference was seen between head orientation and EMG *Path Efficiencies* (p = 0.107) and both were significantly less efficient than the joystick (p < 0.001). A similar trend is seen in terms of *Overshoot* where no detectible difference in seen between head orientation and EMG (p = 0.162), while the joystick exhibited significantly less *Overshoot* that the other two command sources (p < 0.001). Significant differences were seen between all command sources for *Reaction Time*, with head orientation being the slowest (p < 0.001), EMG the fastest, and the joystick in the middle (p = 0.025). This trend is also seen in *Average Speed*, where head orientation is the slowest (p < 0.001), EMG the fastest, and the joystick again in the middle (p = 0.002).

**Table 2:** **Command source performance, ANOVA results, and fixed effects between sources**

**Performance**	**Command source**			**ANOVA**	**Fixed effects, p**	
**Measure**	**Head Or.**	**EMG**	**Joystick**	**p**	**HdOr.**	**Joystick**
*Throughput (bits/s)*	0.27 ± 0.09	0.33 ± 0.13	0.38 ± 0.12	**<2e-16**	**0.004**	0.122
*Path Efficiency*	33% ± 7%	30% ± 8%	40% ± 9%	**1.603e-9**	0.107	**6.94e-5**
*Overshoot*	127% ± 56%	119% ± 74%	74% ± 47%	**3.131e-8**	0.162	**4.83e-4**
*Reaction Time (s)*	0.42 ± 0.14	0.32 ± 0.13	0.34 ± 0.07	**0.004344**	**6.34e-5**	**0.025**
*Average Speed (m/s)*	0.95 ± 0.22	1.17 ± 0.12	1.03 ± 0.17	**4.83e-7**	**1.19e-4**	**0.002**

Boldface values denote statistical significance.

Table 3 details the other significant interactions between index of difficulty and performance measures and index of difficulty combined with command source. Significant effects of index of difficulty were seen for head orientation (p = 0.043) and the joystick (p = 0.020) *Throughput*, while ID did not significantly impact *Throughput* while using the EMG command source (p = 0.164). Across all sources, *Overshoot* and *Reaction Time* were impacted by index of difficulty (p < 0.001 and p = 0.004 respectively).

**Table 3:** **Fixed effects between performance measures and index of difficulty (ID) and ID combined with command source**

**Performance**	**Fixed effects, p**				
**Measure**	**ID**	ID*Source	ID*HdOr	ID*EMG	ID*Joystick
*Throughput (bits/s)*	0.050	**0.040**	**0.043**	0.164	**0.020**
*Path Efficiency*	0.840	0.846	-	-	-
*Overshoot*	**1.704e-11**	0.410	-	-	-
*Reaction Time (s)*	**0.004**	0.272	-	-	-
*Average Speed (m/s)*	0.090	0.846	-	-	-

Boldface values denote statistical significance.

The effect on performance of moving simultaneously in multiple dimensions was quantified by the various *Dimension Ratios*. Overall summary averages of the various *Dimension Ratios* are listed for each command source and each performance measure in Table 4. Note that a Dimension Ratio of less than 1.0 for all but *Overshoot* means that performance declined as the number of dimensions of control increased, while *a Dimension Ratio* of greater than 1.0 for *Overshoot* meant lower performance as the number of control dimensions increased. Across command sources, as the number of dimensions needed to move to the target increased, the *Dimension Ratio* significantly deviated farther from unity for all performance measures except *Average Speed* (p > 0.059) and Joystick *Reaction Time* when moving from 2D to 3D movements (p = 0.141).

**Table 4:** **Summary of the Direction Ratios for each performance measure across command sources, ANOVA results, and fixed effects between sources**

**DR 1,2**						
**Performance**	**Command source**			**ANOVA**	**Fixed effects, p**	
**Measure**	**Head Or.**	**EMG**	**Joystick**	**p**	**HdOr.**	**Joystick**
*Throughput (bits/s)*	0.49 ± 0.11	0.47 ± 0.18	0.51 ± 0.13	0.247	0.391	0.094
*Path Efficiency*	0.54 ± 0.11	0.53 ± 0.19	0.59 ± 0.14	**0.039**	0.571	**0.021**
*Overshoot*	0.84 ± 0.34	1.14 ± 0.40	1.39 ± 0.57	**0**	**0.000**	**0.003**
*Reaction Time (s)*	0.77 ± 0.48	0.81 ± 0.39	0.84 ± 0.57	0.687	0.543	0.739
*Average Speed (m/s)*	1.01 ± 0.09	0.96 ± 0.11	0.97 ± 0.09	**0.005**	**0.003**	0.561
**DR 1,3**						
**Performance**	**Command source**			**ANOVA**	**Fixed effects, p**	
**Measure**	**Head Or.**	**EMG**	**Joystick**	**p**	**HdOr.**	**Joystick**
*Throughput (bits/s)*	0.34 ± 0.09	0.30 ± 0.12	0.36 ± 0.08	**0.001**	**0.040**	**0.001**
*Path Efficiency*	0.39 ± 0.09	0.38 ± 0.14	0.45 ± 0.10	**4e-04**	0.473	**0.001**
*Overshoot*	1.24 ± 0.52	1.38 ± 0.46	1.36 ± 0.66	0.262	0.087	0.814
*Reaction Time (s)*	0.73 ± 0.53	0.85 ± 0.38	0.82 ± 0.58	0.333	0.111	0.729
*Average Speed (m/s)*	0.98 ± 0.08	0.96 ± 0.15	0.94 ± 0.10	0.111	0.194	0.341
**DR 2,3**						
**Performance**	**Command source**			**ANOVA**	**Fixed effects, p**	
**Measure**	**Head Or.**	**EMG**	**Joystick**	**p**	**HdOr.**	**Joystick**
*Throughput (bits/s)*	0.69 ± 0.11	0.64 ± 0.16	0.70 ± 0.10	**0.002**	**0.026**	**0.003**
*Path Efficiency*	0.72 ± 0.11	0.71 ± 0.20	0.76 ± 0.10	**0.04**	0.744	**0.050**
*Overshoot*	1.48 ± 0.46	1.21 ± 0.45	0.98 ± 0.30	**0**	**0.000**	**0.000**
*Reaction Time (s)*	0.95 ± 0.40	1.05 ± 0.39	0.98 ± 0.41	0.208	0.082	0.244
*Average Speed (m/s)*	0.98 ± 0.09	1.00 ± 0.08	0.97 ± 0.09	**0.05**	0.053	**0.009**

Boldface values denote statistical significance.

In moving from 1D to 2D movements (*DR 1,2*), both 2D *Throughput and Path Efficiency* are about half that of 1D movements with significant differences between command sources present in the *Path Efficiency*, *Overshoot*, and *Average Speed* (Table 4). Within these performance measures, Head Orientation exhibited a significantly different effect on *Overshoot* due to dimension of movement than EMG (p < 0.001) and Joystick (p = 0.003) and less effects on *Average Speed* compared to EMG (p = 0.003). Compare to EMG, thee joystick exhibited significantly less impact due to increased dimension of movement (going from 1 to 2 dimensions) on *Path Efficiency* (p = 0.021) but a greater effect on *Overshoot* (p = 0.003).

The trend of reduced performance due to increased dimension of movement continued when comparing 1D and 3D motions, particularly in *Throughput* and *Path Efficiency* across command sources (Table 4). The *Dimension Ratio* between 1 and three dimensions (*DR 1,3*) showed significant differences only in *Throughput* and *Path Efficiency* (Table 4). All command sources exhibited a different *Throughput DR 1,3* (p = 0.001) while only a significant difference between the EMG and Joystick *Path Efficiencies DR 1,3* was observed (p = 0.001).

Significant differences between command sources were seen in all *Dimension Ratios* when moving from 2 to 3 dimensions (DR 2,3) except *Reaction Time* (p < 0.05) (Table 4). Both the Head Orientation *Throughput* and *Overshoot DR 2,3* were significantly greater (p < 0.026) than those for EMG. In this *Dimension Ratio*, the joystick was significantly less impacted by the increased dimension (p < 0.05) than EMG or Head Orientation in all performance measure except *Average Speed*.

## Discussion

This study investigated the performance of three user interfaces for controlling a robot arm in three dimensions, two of which (head orientation and face/neck EMG) could serve as an effective command source for individuals with a high cervical spinal cord injury, and one (three axis joystick) that served as a standard of comparison. While all three interfaces exhibited somewhat similar performance and operation, the joystick was marginally superior to head orientation and EMG, exhibiting slightly better accuracy and faster speed and response time. This was expected given the familiarity of the interface and the highly dexterous nature of the hand and arm, however, the fact that head orientation and EMG control (both of which can be used by individuals with high cervical SCI) were only slightly inferior in performance indicates that such an individual should be able to use these interfaces to operate a service robot or their own arm that has been reanimated using a FES-based neuroprosthesis almost as capably as an able-bodied individual using a joystick.

To address whether fatigue or learning effects were present during the trials, command source performance was assessed using experimental blocks two and three of the three block experiment. The performance was found to be consistent between these blocks with no statistical difference in performance (p > 0.05) for all subjects demonstrating that no learning or fatigue effects were observed over this portion of the experiment.

### Effect of dimensionality

The relative similarity in performance between head orientation and EMG (and the joystick as well) is largely due to the manner in which subjects used the interfaces in our multi-dimensional task. Subjects adopted a sequential mode of operation, moving first along one axis, then another, and then another to the target, with few “diagonal” movements requiring simultaneous control of both movement directions. This is particularly true given the low *Path Efficiencies* observed. Subjects exhibited some simultaneous two-axis movements as seen in Figure 3, but never simultaneous motion of all three movement dimensions. This behavior was seen for all three command sources, and probably reflects the requirement to control a set of three movements with three different and mechanically unrelated movements. This matches the performance seen by both Tijsma [7], and Zhai [22] who showed that users rarely operate 3D user input devices in multiple degrees of freedom to complete a 3D task and when expressly told to do so, will sacrifice time to improve coordination between degrees of freedom.

While this study controlled for short-term learning effects with the initial practice block, it is not known if simultaneous control of command actions (and hence, performance) would improve with long-term practice. No studies (this one or those previously mentioned) have explored the effects of long-term practice on 3D user interfaces. It is possible that, similar to other motor tasks, users will become more accustomed to the interface and the actions required will become more “natural”, and that simultaneous control will thus also improve.

### EMG interface limitations

It should be noted that given the number of muscles (four) and algorithm used, true 3D motion using EMG was not possible because some commands would counteract others. For example, it would be impossible to simultaneously move outward, down, and to the right. The initial decision to use four muscles was set by the number of EMG channels available in the clinical device this work was supporting, as well as the number of distinct muscles/actions available to an individual with a cervical spinal cord injury. This was not a likely limitation to performance, however, given how the command source was used in a sequential manner. It is possible that an expanded muscle set would improve EMG performance by not requiring the use of co-contraction for forward/backward control, e.g., if six muscles were available. This may also reduce the differences noticed between the forward and backward EMG performance. At the same time, by having more options, the cognitive load on the user might increase, particularly for a signal like EMG with its coarse resolution of control. Another possibility with an expanded muscle set would be to use EMG to estimate head motions from head actuator muscles. This approach has been investigated in the past with mixed results [27,28] largely due to the need to use relatively large (>45°) head rotations for classification. The use of implanted electrodes, however, may improve performance as the electrodes will be attached to the muscles themselves, and will not be affected by skin movement as in the case of neck surface EMG.

The fact that two of the eight subjects participating in the EMG experiments could not independently control the left and right platysma muscles followed the trend seen in previous work by the authors [24]. Other sets of candidate muscles were not tested here to determine if this effect is peculiar to the platysma muscle, but that would be an obvious next step. If such lack of independent control is found in other sets of control muscles, this could limit the use of face and neck EMG signals as a command source in some individuals.

The processing methods used in this study were similar to the standard techniques currently in use by EMG controlled prosthetic systems (e.g., functional electrical stimulation and upper extremity prosthetics). While additional features can be extracted from EMG signals using other signal processing techniques, many features of the EMG signal are generally correlated to the rectified, windowed, average EMG amplitude. Additional pattern recognition techniques may be employed in future studies to further enhance the performance of this command source.

### Impact of limiting command velocity

Figure 4C shows that some speed limiting occurred in EMG commanded movements as a result of the command speed being limited to 300 mm/s. Some minor speed limiting was also observed in the head orientation and joystick command sources, but this was generally negligible. In preliminary testing, subject performance dropped significantly when the EMG command speed limit was raised above 300 mm/s, largely due to increased *Overshoot* and reduced velocity control. For this reason, the maximum command speed was set to limit *Overshoot* to a reasonable level while still providing sufficient speed to reach far targets in a timely manner. While allowing higher command velocities may have allowed a higher overall *Throughput* for EMG-commanded end-point motions, it is more likely that a corresponding increase in *Overshoot* would have reduced performance to an even greater extent. It is also possible that, with experience though long term practice, that the speed limit could be increased for clinical users. It is not believed, however, that limiting command velocity significantly impacted the computed measures of performance in our study. The high amount of velocity limiting is also due to the command algorithm mapping maximum endpoint speed to sub-MVC (~70% MVC) levels of contraction. While this was done to reduce the overall effort of using the interface and prevent fatigue, it did allow participants to produce brief EMG commands that “spiked” the velocity to its maximum. While this does occur, the bulk of the commands from the user interfaces were controlling slow movements such that low velocity commands dominated the overall performance measures. Subjects tended to start with a high velocity burst to move toward the target volume and then smaller, low speed commands to reach the target. This afforded better control and minimized the amount of maximum or near maximum muscle contractions required to perform the task.

### Fitts’ Law for head orientation-commanded and EMG-commanded 3D end-point movements

From Figure 5A and (B), it can be seen that the linear fit between the index of difficulty (ID) and movement time (MT) of the head orientation and EMG data to a Fitts’ Law model was certainly not perfect, with R^2^ values of 0.45 and 0.46, respectively. The joystick followed an ideal Fitts’ Law model somewhat better, with an R^2^ of 0.70 - due primarily to its higher *Path Efficiency* and lower *Overshoot* compared to the other two command sources. However, this does not rule out the use of the Fitts’ Law based *Throughput* performance measure. In previous 2D studies by the authors and others, it was observed that the smallest diameter targets resulted in disproportionately long movement times that did not always follow a Fitts’ Law model of movement [24,26]. This was also seen in this study in the significant effect of index of difficulty on *Overshoot* (Table 3), with more *Overshoot* requiring an increased time to re-acquire the target. When these targets were removed from the regression for our data, the linear fit achieved a significant (p < 0.05) R^2^ of 0.69, 0.83, and 0.91 for head orientation, EMG, and joystick commanded motion, respectively. The deviations we observed are thus most likely a result of the original characterization of Fitts’ Law to directed, continual, position commanded movement. When the command source is a velocity command (as in the case of the command sources used in this and other human-computer interface studies) and some targets are too small to acquire in a single trajectory due to excessive *Overshoot*, a strict fit to Fitts’ Law is often not observed [17,26,29]. Furthermore, the use of sequential dimension commands seen in all three command sources contrasts with the smooth, ballistic trajectory to the target seen in classic Fitts’ Law tasks, and limits the interpretation of *Throughput* as an overall summary performance measure. However, the use of additional performance measures such as *Path Efficiency*, *Overshoot*, *Reaction Time* and *Average Speed*, provide ample detail concerning the finer aspects of command source performance in a way that does not depend on the Fitts’ Law model [18,24]. While it is understood that a Fitts’ Law model does not describe the motion as observed, it was included in the analysis for comparison to other user interface studies and to highlight the importance of the utility of using additional, non-summary performance measures to better assess command source performance.

### Head orientation versus EMG as a command source

Overall, the performance of the robotic command interface based on neck-face EMG was marginally better than that of head orientation in commanding 3D motions. While *Path Efficiency* and *Overshoot* may be similar between the two command sources, EMG had a significantly higher *Throughput*, faster *Reaction Time*, and faster *Average* Speed than that of head orientation. However, EMG also exhibited a much higher number of *Z Commands* as a result of subjects using several bursts of activity to command movement in that direction rather than a single smooth command, i.e., users employed a more binary (on/off) operation for vertical end-point control via the EMG interface.

The difference in performance of the head orientation and EMG-based interfaces in the 3D control of a robot arm is in contrast to that seen in 2D computer cursor control with the same command sources [23], where head orientation was shown to be superior in terms of *Path Efficiency* (88 ± 2% vs. 61 ± 10%) and *Overshoot* (32 ± 8% vs. 83 ± 40%) and with no significant differences in *Throughput or Reaction Time*. The Direction Ratios’s of head orientation in the earlier 2D cursor control study were also much closer to unity (i.e., fewer directional effects) than EMG control – which typically exhibited the one-direction-at-a-time command structure observed in the current study for a 3D task. It seems likely that the EMG interface (even for the 2D task) is moderately more demanding on the user than the head orientation interface and that a typical user strategy for addressing more complex interfaces is to revert to a sequence of single dimension commands. Thus, EMG control may have been largely sequential for even the 2D cursor task because it had already reached the threshold for complexity, whereas head orientation did not reach this threshold until the task increased from 2D to 3D tasks.

Given that EMG performed similar or better than head orientation as a user interface for controlling 3D end-point for many of our performance measures, EMG is an intriguing choice as a command source because of other, potentially more practical reasons. In particular, EMG recording systems are already are being implanted in human subjects for the control of neuroprosthesis [9,11,30] and are being investigated for control of powered prostheses following amputation [31-33], and the durability and repeatability of such systems have already been demonstrated [9,33]. On the other hand, no implantable orientation sensor currently exists. In operation, an implanted EMG command source could be nearly invisible to outside observers and controlled using subtle and non-fatiguing muscle contractions. Considering that both obtrusiveness and the amount of worn equipment are related to abandonment rates of prosthetic systems [34], an implanted EMG interface may be an attractive and viable means of commanding systems for restoration of 3D arm function for the foreseeable future.

## Conclusion

This project investigated the performance of head orientation and EMG commanded robot end-point motions, both of which are voluntary actions that are typically available to individuals with a high-cervical SCI and could be used to command a rehabilitation robot or their own paralyzed arm (via an FES-based neuroprosthesis). A three-axis joystick was also evaluated for baseline comparisons. Both head orientation and EMG command sources typically exhibited sequential control of individual movement dimensions rather than simultaneous control of two or three movement directions. The joystick interface had marginally better performance overall in terms of movement times, accuracy, and stability. The EMG interface was faster (higher *Average Speed*, lower *Reaction Time*) and had a higher information transfer rate (*Throughput*) than head orientation, but was similar in terms of straightness to target (*Path Efficiency)* and terminal controllability (*Overshoot*). Given these differences and the ability to be readily implanted, EMG is a practical choice for a 3D command source for controlling an implanted neuroprosthesis or other multi-dimensional assistive devices. The evaluation approach used here should be useful for future evaluations of other 3D user interfaces, including brain-computer interfaces.

### Declarations

#### Ethics approval and consent to participate

Proper informed consent was obtained and pertinent human subject protections were observed, including approval by the Louis Stokes Cleveland VA Medical Center Institutional Review Board (study #2004-050).

## Abbreviations

2D: Two dimensional
3D: Three dimensional
BCI: Brain computer interface
EMG: Electromyography
FES: Functional electrical stimulation
ID: Index of difficulty
MT: Movement time
SCI: Spinal cord injury
